# The effect of trust and proximity on vaccine propensity

**DOI:** 10.1371/journal.pone.0220658

**Published:** 2019-08-28

**Authors:** Florian Justwan, Bert Baumgaertner, Juliet E. Carlisle, Emma Carson, Jordan Kizer

**Affiliations:** 1 Department of Politics and Philosophy, University of Idaho, Moscow, Idaho, United States of America; 2 Department of Political Science, University of Utah, Salt Lake City, Utah, United States of America; 3 Independent Researcher, Moscow, Idaho, United States of America; 4 Lyndon B. Johnson School of Public Affairs, University of Texas, Austin, Texas, United States of America; University of Campania, ITALY

## Abstract

The main goal of this paper is to study the effects of (1) trust in government medical experts and (2) proximity to a recent disease outbreak on vaccine propensity. More specifically, we explore how these variables affect attitudes with regards to measles. Using original survey data, collected in January/February 2017, we obtain three main empirical findings. *First*, contrary to our expectations, an individual’s proximity to a recent measles outbreak has no independent effect on vaccination attitudes. *Second*, corroborating previous studies in the field, we find that trust in institutions such as the CDC has a positive effect on our dependent variable. *Third*, there is a significant interactive relationship between proximity and trust in governmental medical experts. While distance from a previous measles outbreak has *no* effect on vaccination attitudes for respondents with medium or high levels of trust, the variable exerts a negative effect for subjects with little confidence in government medical experts. In other words: low-trust individuals who live farther away from a recent measles outbreak harbor less favorable views about vaccination for this particular disease than low-trust respondents who live close to an affected area. This implies that citizens who are skeptical of the CDC and similar institutions base their vaccination decision-making to some degree on whether or not a given disease occurs in close vicinity to their community.

## Introduction

Before the first measles vaccine was licensed in 1963, the number of reported cases in the U.S. ranged in the hundreds of thousands per year. By the 1980’s the number of cases dropped to 1–10 thousand per year. When the number of cases dropped below a thousand per year by 2000, measles was declared eliminated in the U.S. [1].

Despite the success of vaccination against measles, or perhaps in part because of its success and the corresponding absence of threat of infection, there is growing vaccine hesitancy both in the U.S. [2] and globally [3]. Vaccine hesitancy can manifest itself in increased non-medical exemption rates, which lead to decreases in vaccination rates, which then allows for the possibility of outbreaks, something that has explicitly happened in the U.S. While some states have instituted policies to combat lower vaccination rates by prohibiting non-medical exemptions (e.g. California), 18 states still allow for non-medical exemptions.

Even without exemptions, however, evidence suggests that outbreaks themselves can increase perceptions of risk and thereby decrease vaccine hesitancy. As a result of recent measles outbreaks, the variability of risk perception in terms of both time and space becomes salient. Indeed, scholars have found significant effects between proximity to disease outbreaks and vaccination behavior. For example, following a measles outbreak in California from 1988–1990, Dales et al. [4] find that the strongest vaccination response occurred where media coverage was highest and responses can decay with both time and distance [5][6][7]. In this research we use a nationally representative U.S. sample to systematically explore geographical distance from disease outbreak in relation to vaccination behavior.

The formation of attitudes about vaccination is complex. Research demonstrates vaccine hesitancy being linked to oversight [8], media and peer group influence [9], fears over the number of vaccinations per doctor’s visit [10], distrust of vaccine benefits and science [11], socio-economic barriers (that often interact with race/ethnicity) [12], conscious decisions (for some the result of parental concern over vaccine safety, see Shawn and Gold [13]) and efficacy [14][15]. Research on vaccinations also links vaccination rates to resource and information access [16][17][18]. Furthermore, vaccination rates are dependent upon parental access to vaccine information, including safety and general knowledge.

Despite increases in reported cases of preventable disease outbreaks, many people still refuse vaccinations. Aside from the factors identified above, we believe that in part, vaccine behavior could be linked to proximity to disease outbreaks. This belief is motivated by both theoretical and empirical work on the relationship between proximity and risk perception. Construal Level Theory (CLT), developed by Liberman and Trope [19], specifies four key dimensions of distance in terms of their mental construal: 1) spatial or geographical distance; 2) temporal distance; 3) distance between the perceiver and a social target, i.e. another individual or group; and 4) hypotheticality, e.g., how certain it is that an event will happen. Events that are psychologically more distant are represented by more abstract high-level construals, whereas events that are psychologically close are represented with more concrete low-level construals that include specific contextual details [20]. Moreover, these dimensions are closely linked, so that remote locations will tend to bring to mind the distant future rather than the near future, and unlikely events rather than likely events [19]. Empirical work in environmental and technological risk has demonstrated an association between risk perception and proximity to environmentally-related health threats, such as water pollution [21], nuclear sites [22][23], and lead exposure [24]. In addition, Chandran and Menon [25] have demonstrated that every day frames make risks seem more proximal and concrete than every year framing, thereby affecting self-risk perceptions. Given the breadth of this work, we also expect to find an association between proximity and risk perception in the case of infectious diseases.

In this spirit, several empirical studies demonstrate a link between geographic proximity to virus outbreaks and perception of risk. The link, though complicated, is consistent with the expectation that perception of risk increases as (psychological) distance decreases. For example, Rudisill et al. [26] find that individuals near avian flu outbreaks perceive their risk of contraction as higher and respond by reducing their consumption of poultry and fowl goods. This effect is moderated by knowledge/information; learning facts regarding contagion and prevention decreased the likelihood to exhibit consumption behaviors. Moreover, an individual's proximity to outbreaks influenced their reception of the information and thus individual risk perceptions, and the effect of knowledge is diminished when human cases of avian flu occur in close proximity. In studying West Nile virus in North America, Zielinski et al. [27] find that as individuals gain access to information about the disease and its proximity, their perception of the risks associated with contraction and the disease grow. Jena and Khullar [28] similarly find that proximity to pertussis outbreaks was associated with an increase in vaccinated children in the county of the outbreak. This increase is presumably the effect of increased perception of risk–in contrast to the expected increases in vaccination in California, where institutional changes no longer allow non-medical exemptions. Along the same lines, Rosoff, John, and Prager [29] find that the closer a simulated flu outbreak was reported to be, the greater the risk perception of the respondent. However, they also find that the cause of the outbreak, e.g. a terrorist attack or environmental accident, mattered—if individuals perceive that foul-play and human agency is involved, they believe that they are at less risk of contraction, despite the probability of contraction being fixed.

In sum, current research suggests that risk perception can be explained by psychological proximity to outbreaks: individuals respond more strongly to risks when those risks are of low construal—specific, concrete, proximate. This generalization appears to hold across various health-related contexts. To our knowledge, however, no tests have been done to determine whether this relationship holds with geographic proximity to recent measles outbreaks (2016 specifically). Our research herein considers the relationship between geographic proximity and vaccination behavior. We thus have the following hypothesis:

**Hypothesis 1.** Individuals who live close to a recent outbreak of measles are more likely to get vaccinated than individuals who live farther away.

As the literature above suggests, there are nuances to the relationship between proximity and risk perception and it is not expected that proximity alone explains why some individuals choose to vaccinate and others do not. We are additionally interested in the role of trust. Broadly, trust in government, science, and medical officials have been shown to affect risk perceptions with regards to environment and health issues [30][31][32]. Previous studies suggest that individuals who distrust government medical officials will adversely perceive the threat posed by issues such as climate change, diseases, pollution, or vaccines. Trust in government medical officials has also been found to have implications for vaccination, such that increased levels of trust in government medical experts positively impact vaccination rates [33][31]. Confidence in organizations such as the Centers for Disease Control and Prevention (CDC) is an important predictor of vaccination behavior since these entities provide consistent and positive cues to citizens about issues related to vaccine safety, vaccine risk, and vaccine effectiveness. As a result, “the less people trust […] scientific institutions the more likely they are to believe a link between vaccines and autism and thus, the less likely they are to demonstrate support for vaccinations” [34].

As far as we know, very little research exists on the interplay between trust and proximity. One exception is Vaske et al. [32], who examine hunters’ risk perceptions to Chronic Wasting Disease (CWD) relative to their county locations and their level of trust in the government managing-agency. They find that hunters residing in counties where CWD was present perceived less risk in contracting the disease. This can be explained by lower levels of trust in the managing agencies. Level of trust in officials can distort risk perceptions by making individuals less likely to take managing agency information seriously.

Synthesizing these considerations regarding trust and its intersection with proximity, it seems reasonable to suppose that variation in proximity to disease outbreaks will have larger effects on vaccination attitudes for those with lower levels of trust in government medical experts (GMEs) than for those with higher levels of trust. If a given individual has high confidence in institutions such as the CDC, this person is likely to fully internalize the pro-vaccination cues provided by this entity regardless of whether this person lives close to a recent measles outbreak. By contrast, respondents with low levels of trust in government medical experts are unlikely to adopt categorical pro-vaccination views. Instead, these individuals will tend to discount the provided information about the dangers of the disease and the effectiveness of the vaccine. As a result, people with low levels of trust should be more responsive to their personal risk environment and, as such, base their vaccine decision-making on how proximate the disease is to themselves. The recent (2016) outbreaks of measles allows us to test these considerations. This leads us to formulate the following hypothesis:

**Hypothesis 2.** Individuals with lower levels of trust in government medical experts will exhibit more variation in their vaccination attitude as a function of proximity to measles outbreaks.

Our research supplements current findings by suggesting that individuals who are less trusting of government officials and distantly located from outbreaks will be less likely to favorably view vaccines and be less likely to vaccinate. The body of literature suggests that while many factors play into an individual’s risk perceptions, proximity and trust are critical in areas relating to health issues.

## Methods

### Data collection and sample characteristics

Our statistical analysis is based on data from an original online survey. University of Idaho granted Institutional Review Board exemption for this research endeavor under category 2 at 45 Code of Federal Regulations 46.101(b)(2) [Project Number: 17–007]. Data were analyzed anonymously. Respondents were informed about the project and they provided written consent to participate.

Data collection proceeded in three steps. *First*, we designed a questionnaire containing a wide range of items about a respondent’s general demographic characteristics as well as attitudes about vaccinations. *Second*, we programmed our survey on an online platform (Qualtrics) and forwarded the link to the questionnaire to Survey Sampling International (SSI), a market research firm based in Shelton, Connecticut. *Third*, SSI distributed the survey to a nationally representative sample of the U.S. voting age population. SSI generates its respondent pools by recruiting members of its country-specific online panels. In 2017 (the year of our survey), SSI’s U.S. panel contained over 7 million potential respondents [35]. The survey underwent a soft launch (10% completion) in order to review and make minor adjustments. Data were collected between January 25–27, 2017.

The survey consisted of three sections. The first section included a series of questions tapping into respondents’ political beliefs. The second section included questions about vaccination attitudes. Finally, all respondents were asked a series of basic questions about their demographics.

As we show in S1 Table our final sample matches known population parameters in the United States with regards to gender, age, income, race, and Census region. We chose a standard sample size of roughly 1,000 respondents (n = 1,006) in order to bring our study in line with other observational survey research. In the next section, we discuss the variables that we used in order to test our theoretical expectations.

### Dependent variable

Our dependent variable is an individual’s vaccination attitude with regards to measles. In order to tap into this dimension, we asked our respondents to imagine that they are currently missing the immunization for this particular disease. We then inquired how likely or unlikely they would be to get vaccinated in two different hypothetical scenarios: (a) if there was no immediate risk of getting infected, and (b) if there was an outbreak of the disease in their community. For both scenarios, survey takers could choose from the following answer options: (0) very unlikely, (1) unlikely, (2) neither likely nor unlikely, (3) likely, and (4) very likely.

Prior to conducting the analysis, we expected that our subjects would differ in their responses to the high risk and low risk scenarios. However, this expectation was not supported. Most people (62 percent) gave the same answer to both survey questions. As a result, the items are highly correlated (r = 0.78) and thus clearly tap into the same underlying dimension. Our subsequent statistical analysis is therefore based on one dependent variable in which we calculate an individual’s aggregate score across both survey items discussed above. The variable ranges from 0 to 8. Higher values correspond with more positive views about vaccinations with regards to measles. In S2 Table, we show that our statistical results are substantively identical if we estimate separate models with the two base indicators.

### Main independent variables

One of our two main independent variables is an individual’s proximity to a recent measles outbreak. For our purposes, we define “recent” as occurring within one year prior to our survey. While any time window chosen by a researcher is somewhat arbitrary, this one-year cutoff should ensure that any previously recorded measles outbreak in close proximity to a respondent was still fairly salient at the time of data collection.

According to the Centers for Disease Control and Prevention, there were two measles outbreaks between January 2016 and January 2017 (the start of our survey). The first one occurred in Shelby County, TN between April and May [36]. The second one followed in Eloy, AZ between May and June [37]. Both of these cases were covered extensively by various news outlets. Given the extensive news coverage, there are strong reasons to believe that quite a few respondents in our dataset were aware of these outbreaks–especially if they lived close to any of the affected communities.

In order to capture an individual’s distance from Shelby Country, TN and Eloy, AZ, we proceeded as follows. *First*, as part of our survey, we recorded a respondent’s zip code in our dataset. *Second*, we identified every zip code affected by the two measles outbreaks (60 zip codes in Shelby County, 1 in Eloy, AZ). *Third*, using information from the United States Census Bureau, we assigned geographic longitude and latitude to each zip code in our dataset. It is important to note that the Census Bureau only provides information for “Zip Code Tabulation Areas” (ZCTAs). ZCTAs are based on zip codes, but occasionally diverge from zip codes for the sake of creating more cohesive geographic boundaries. However, in most cases, zip codes and ZCTAs are equivalent (United States Census Bureau, 2010). When they do diverge, the error introduced is minimal. *Fourth*, we calculated straight-line distances (in miles) between each respondent’s own zip code and the measles-affected zip code closest to a given individual. We opted to calculate straight-line distances rather than driving distance or other methods for two reasons: (1) straight-line distances ensure consistency of data regardless of geographic region, (2) travel distance has little bearing on information sharing between individuals. The final distance-variable ranges from 0 to 2946, with a mean of 586.36 and a standard deviation of 335.37.

According to our second hypothesis, the effect of proximity should be moderated by an individual’s level of trust in government medical experts. We measured this variable by asking our respondents how much they “trust government medical exerts (such as Centers for Disease Control and Prevention) regarding questions about health.” Subjects could choose one of six response options: (1) strongly distrust, (2) somewhat distrust, (3) neither trust nor distrust, (4) somewhat trust, (5) strongly trust, and (6) I don’t know. All respondents in the last category (“I don’t know”) were excluded from the subsequent analysis. Below, we treat trust as a nominal variable (baseline: neither trust nor distrust). This approach has a number of important advantages. First, it will allow us to obtain more fine-grained insights about the relationship between this correlate and vaccination attitudes than if we treated it as an ordinal predictor. Second, model fit is improved significantly (p<0.05) if we follow this procedure.

### Control variables

We add a number of control variables to account for other causes of micro-level differences in vaccination attitudes. Given that there are strong theoretical reasons to expect that respondents with children hold different views about vaccinations than other individuals, we control for whether or not a given survey taker has any “children or dependents for whom they make medical decisions.” We expect that people with children (coded as “1”) will score higher on our dependent variable than those without since they are more sensitized to the danger of measles. In addition, we account for a variable that captures how closely a respondent follows news and current events. More specifically, we ask our subjects how many days they “watch, read, or listen to the news” during a typical week (excluding sports). We expect that this variable has a positive effect since individuals who pay close attention to news and current events should be more informed about recent outbreaks of various infectious diseases (including, but not limited to measles). Finally, we also control for a series of standard demographic variables: age, gender, education, income, and race (White 1/0). Summary statistics for all variables in this paper can be found in Table 1.

**Table 1:** Descriptive statistics.

Variable	Min	Max	Mean	Std. Dev.	Number of responses	Missing (incl. dk)
*Dependent Variable*						
Attitude about Vaccinations for Measles	0	8	5.46	2.57	965	41
*Independent Variables*						
Proximity to Recent Measles Outbreak	0	2,946	586.36	335.37	1,006	0
Age of Respondent (in years)	18	97	46.5	16.3	997	9
News Consumption (days per week)	0	7	5.20	2.15	992	14
Trust in Government Medical Experts	1	5	3.57	1.04	989	17
1: Strongly Distrust					48	
2: Distrust					95	
3: Neither Trust nor Distrust					265	
4: Trust					409	
5: Strongly Trust					172	
R. Makes Med. Decisions for at Least one Child	0	1	0.33	0.47	996	10
1: Yes					327	
0: No					669	
Gender of Respondent	0	1	0.47	0.49	1,001	5
1: Male					474	
0: Female					527	
Education Level of Respondent	1	8	5.02	1.69	1,005	1
1: Less than High School					2	
2: Incomplete High School					21	
3: High School Graduate					190	
4: Some College, No Degree					255	
5: Two Year Associate Degree					117	
6: Four Year College or Univ. Degree					239	
7: Some Postgraduate School					46	
8: Postgraduate / Professional Degree					135	
Income Level of Respondent	1	12	6.17	3.41	1,005	1
1: Less than $10,000					58	
2: $10,000 - $19,999					110	
3: $20,000 - $29,999					113	
4: $30,000 - $39,999					108	
5: $40,000 - $49,999					91	
6: $50,000 - $59,999					96	
7: $60,000 - $69,999					78	
8: $70,000 - $79,999					74	
9: $80,000 - $89,999					38	
10: $90,000 - $99,999					49	
11: $100,000 - $149,999					125	
12: More than $150,000					65	
Race of Respondent	0	1	0.75	0.43	1,006	0
1: White					758	
0: Non-White					248	

### Analytical approach

All data recoding and statistical analyses were conducted using Stata 14. For any new variables generated, we tested for consistency with the original variable, and stored them as new variables along with the original. To test our hypotheses we used an ordered logistic regression model. The ordered logistic regression is recommended when the dependent variable is ordinal (in our case, the likelihood of getting vaccinated). We present two models to test our hypotheses. Model 1 includes the effects of each independent variable, including proximity to the measles outbreak and trust in government medical experts along with our control variables. In Model 2 we add-in interaction terms (proximity to the measles outbreak x trust in government medical experts). The limit for statistically significant differences was set at p≤0.10.

## Results

Results from our statistical analyses are presented in Table 2. According to our findings in Model 1, an individual’s proximity to a recent measles outbreak has *no* independent effect on vaccination attitudes. The coefficient for this variable is positive but statistically insignificant (B = 0.001; std. error: 0.001; p = 0.43). These results run counter to our theoretical expectations and they suggest that most respondents in our dataset are *un*affected by proximity. Results are different for our other main variable of interest. We find that trust in government medical experts is strongly and positively related to vaccination attitudes. More specifically, respondents who “strongly trust” (B = 1.340; std. error: 0.197; p = 0.01) and “somewhat trust” (B = 0.530; std. error: 0.144; p = 0.01) organizations such as the CDC are significantly more likely to hold favorable views about vaccination for measles than respondents in the base category. By contrast, citizens at the low end of the trust spectrum tend to be skeptical about immunizations (B = -0.966; std. error: 0.309; p = 0.01). These findings are in line with previous research by Baumgaertner et al. [34].

**Table 2:** Ordered logistic regression of vaccination likelihood.

	Model 1	Model 2
**Age of Respondent (in years)** (Min = 18 / Max = 97)	-0.017** (0.004)	-0.018** (0.004)
**Gender of Respondent** (Female = 0 / Male = 1)	-0.202* (0.120)	-0.217* (0.121)
**Education Level of Respondent** (Min = 1 / Max = 8)	0.106** (0.040)	0.110** (0.040)
**Income Level of Respondent** (Min = 1 / Max = 12)	0.029 (0.020)	0.027 (0.020)
**Race of Respondent** (Non-White = 0 / White = 1)	0.166 (0.146)	0.170 (0.146)
**R. Makes Medical Decisions for at Least one Child** (No = 0 / Yes = 1)	0.505** (0.134)	0.518** (0.135)
**News Consumption of Respondent (days per week)** (Min = 0 / Max = 7)	0.088** (0.031)	0.088** (0.032)
**Respondent Proximity to Recent Measles Outbreak** (Min = 0 / Max = 2,946)	0.001 (0.001)	0.001 (0.001)
**Trust in Gov. Medical Experts (“Strongly distrust”)** (Strongly Distrust = 1 / Other = 0)	-0.966** (0.309)	0.586 (0.639)
**Trust in Gov. Medical Experts (“Somewhat distrust”)** (Somewhat Distrust = 1 / Other = 0)	0.002 (0.225)	0.613 (0.493)
**Trust in Gov. Medical Experts (“Somewhat trust”)** (Somewhat Trust = 1 / Other = 0)	0.530** (0.144)	0.704** (0.273)
**Trust in Gov. Medical Experts (“Strongly trust”)** (Strongly Trust = 1 / Other = 0)	1.340** (0.197)	1.381** (0.380)
**Proximity X Strongly distrust**	—	-0.003** (0.001)
**Proximity X Somewhat distrust**	—	-0.001 (0.001)
**Proximity X Somewhat trust**	—	-0.001 (0.001)
**Proximity X Strongly trust**	—	-0.001 (0.001)
**Cut Point 1**	-1.558 (0.319)	-1.402 (0.350)
**Cut Point 2**	-1.246 (0.317)	-1.087 (0.344)
**Cut Point 3**	-0.703 (0.307)	-0.539 (0.339)
**Cut Point 4**	-0.427 (0.305)	-0.259 (0.337)
**Cut Point 5**	0.359 (0.304)	0.536 (0.337)
**Cut Point 6**	0.712 (0.305)	0.893 (0.338)
**Cut Point 7**	1.305 (0.307)	1.489 (0.341)
**Cut Point 8**	1.987 (0.311)	2.172 (0.345)
**Number of Observations**	919	919
**Log-Likelihood**	-1711.23	-1.706.75

* = p ≤ 0.10

** = p ≤ 0.05

In Model 2, we interacted the two correlates discussed above. There are four interaction terms, each one testing whether the effect of proximity varies between people with different levels of confidence in government medical experts. A likelihood ratio test suggests that Model 2 fits the data better than Model 1 (p<0.10). In particular, we see that one of the four interaction terms (Proximity X Strongly distrust) is statistically significant (B = -0.003; std. error: 0.001; p = 0.01). This demonstrates that an individual’s distance to a recent measles outbreak has a different effect for people with low levels of trust in government medical experts than for respondents who “neither trust nor distrust” organizations such as the CDC.

**Fig 1:**
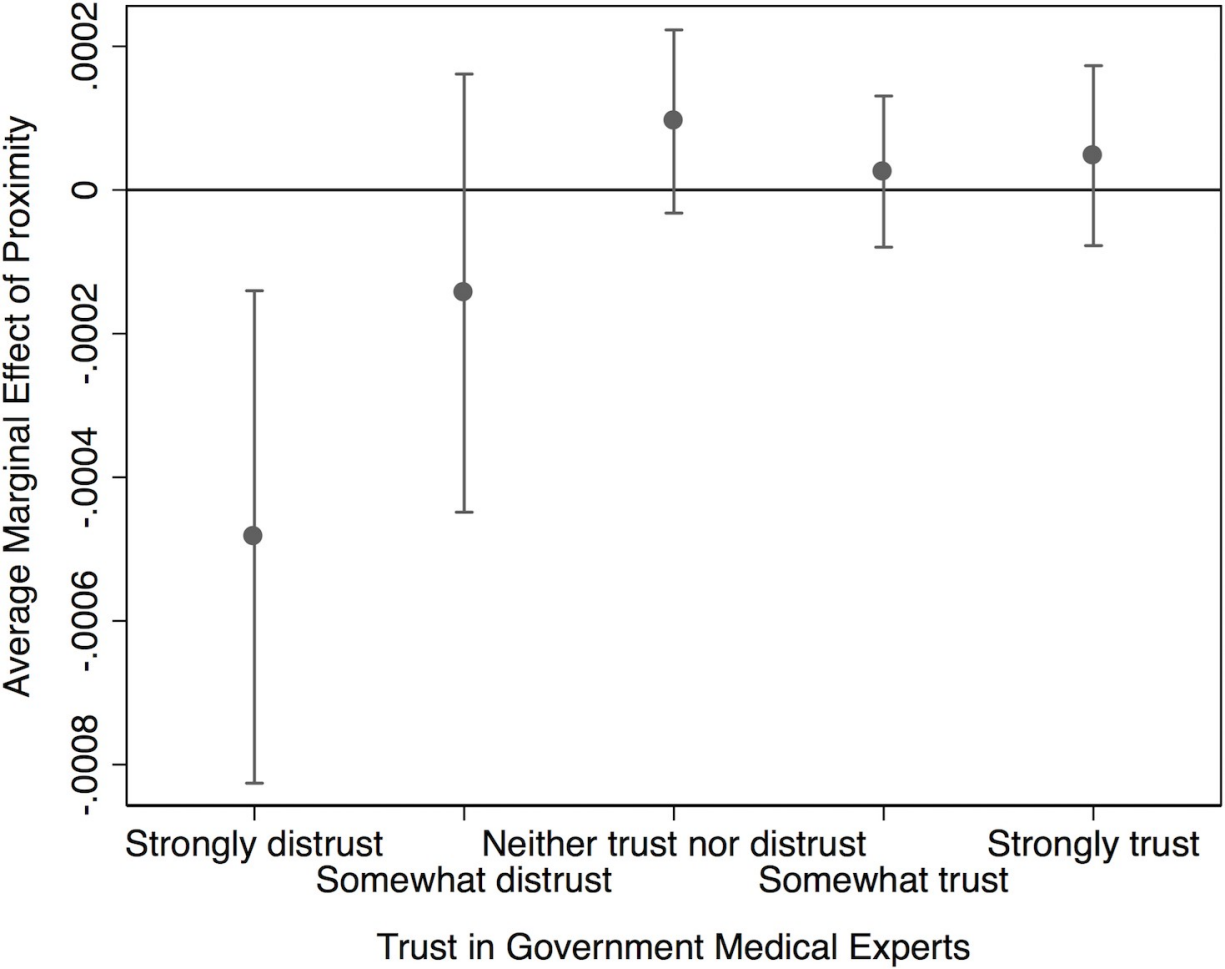
Effect of proximity by trust in GMEs.

Substantive results for interaction terms are best displayed graphically. Fig 1 plots the average marginal effect of distance from a recent measles outbreak at various levels of trust. We see that proximity has *no* influence on vaccination attitudes for respondents with medium or high levels of trust. However, for subjects with little confidence in government medical experts, distance is negatively related to our dependent variable. In other words: low-trust individuals who live farther away from a recent measles outbreak harbor less favorable views about vaccination for this particular disease than low-trust respondents who live close to an affected area. This implies that citizens who are skeptical of the CDC and similar institutions base their vaccination decision-making to some degree on whether or not a given disease occurs in close vicinity to their community. These empirical relationships offer clear evidence in support of Hypothesis 2.

**Fig 2:**
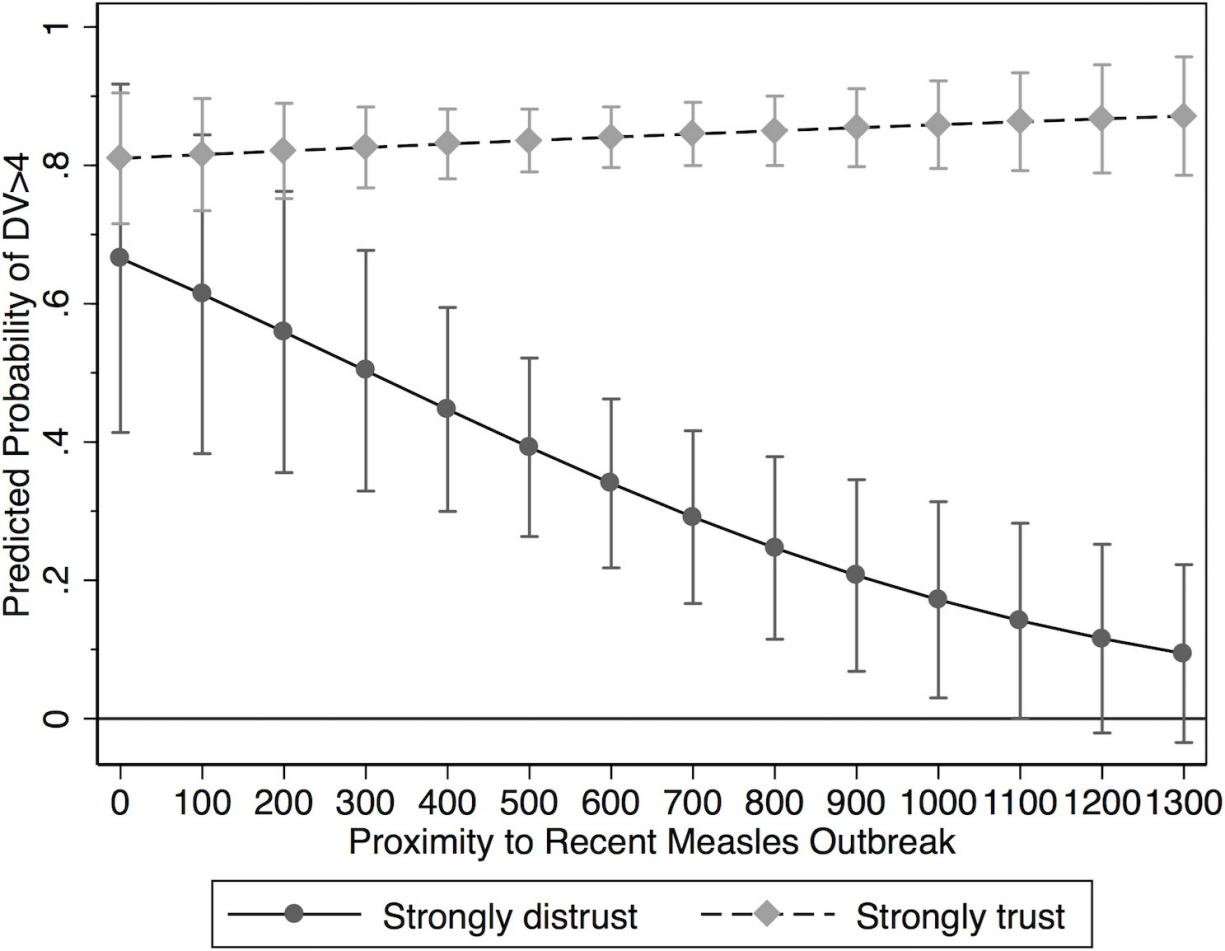
Predicted score.

In order to obtain a deeper understanding of the interactive relationship uncovered in Model 2, we calculated predicted probabilities for both high- and low-trust individuals at different levels of proximity. More specifically, we estimated the probability that a given respondent falls above the midpoint of our vaccination attitude scale (i.e., that s/he scores a 5, 6, 7 or 8 on our dependent variable). A summary of these calculations is provided in Fig 2. Holding all other variables at their observed values, a high-trust respondent who lives in a community affected by measles (that is, a survey taker with a proximity value of “0”) has a predicted probability of 81.0 percent. In other words, this person is expected to indicate that s/he would be fairly likely to vaccinate against measles if s/he lacked the immunization for that disease. For a respondent with low confidence in government medical experts, the corresponding value is 66.6 percent. While this second estimate is somewhat lower than the first, both values are statistically indistinguishable from each other. In other words, there is no meaningful difference in vaccination attitudes for high- and low-trust respondents who live in an area affected by measles.

This picture changes quickly at increasing levels of proximity. For subjects with high levels of trust, the predicted probability remains fairly constant. At an observed distance of 100 miles, the estimate is 81.6 percent. At 500 miles, the value is 83.6 percent, at 1,000 miles it is 85.9 percent, and at 1,300 miles (roughly 2 standard deviations from the mean) it is 87.1 percent. None of these projections are statistically different from each other. For low-trust individuals however, the prediction decreases rather steeply (and significantly) at increasing values of proximity. The relevant probability estimates are 61.4 percent (100 miles), 39.2 percent (500 miles), and 17.2 percent (1,000 miles). At 1,200 miles from a recent measles outbreak, a person’s projected probability of falling into the upper segment of our vaccination propensity scale is statistically indistinguishable from 0. In sum, these findings provide strong evidence in support of Hypothesis 2, and they suggest that respondents who have little confidence in organizations such as the CDC are unlikely to vaccinate against measles if the disease is spatially distant.

A number of control variables are statistically significant as well. First, we see that age is negatively related to vaccination attitudes about measles (B = -0.017; std. error: 0.004; p = 0.01). This likely reflects decreased risk perception by older individuals in our dataset. Second, higher levels of education are associated with more favorable views about vaccinations (B = 0.110; std. error: 0.040; p = 0.01). Finally, as expected, individuals with children (B = 0.518; std. error: 0.135; p = 0.01), women (B = 0.217; std. error: 0.121; p<0.10) as well as subjects who follow news very closely (B = 0.088; std. error: 0.032; p = 0.01) score higher on our dependent variable than other respondents. This suggests that vaccination attitudes are at least in part driven by people’s social and informational environments.

In a final step, we compare substantive effect sizes for all significant variables in Model 2. Results are summarized in Table 3. Similar to above, we estimated the probability that a given respondent falls above the midpoint (“4”) on our dependent variable. For each correlate, we generated two estimates: the probability at the minimum value of a given control variable and the corresponding value when the item is at its maximum. Holding all other predictors at their observed values, an 18-year-old respondent in our dataset has a 76.3 percent probability of scoring a “5” or higher on our dependent variable. In other words, very young individuals are quite likely to indicate that they would vaccinate against measles if they missed the relevant immunization. By contrast, for a 97-year-old individual, the estimated value is only 48.6 percent. Put differently, across the full range of our age variable, the predicted probability of holding favorable views about the measles vaccine decreases by 27.7 percent. Next, we assess the effect of gender. The substantive impact of this variable is much smaller. Compared to men (probability: 64.9 percent), women are only slightly more likely to fall into the upper segment of our dependent variable (probability: 69.2 percent). Nevertheless, this finding suggests that there are slight differences in how men and women think about vaccinations with regards to measles. Next, education, news consumption, and having young children all have positive effects on our dependent variable. In particular, we find that individuals with postgraduate degrees are about 15.5 percent more likely to express favorable vaccination attitudes than respondents who have not attended high school. Likewise, survey takers who have children for whom they make medical decisions have a 10 percent higher probability of holding positive views about immunizations than other subjects. Lastly, individuals who never “watch, read, or listen to the news” receive a predicted probability of 57.6 percent. By contrast, the estimated margin for the most active news consumers is 70.2 percent.

**Table 3:** Comparison of predicted probabilities.

	Predicted Probability *Dependent Variable Score >4*		
	Min of Var.	Max of Var.	Δ
**Age** (Min = 18 / Max = 97)	76.3%	48.6%	- 27.7%
**Gender** (Female = 0 / Male = 1)	69.2%	64.9%	- 4.3%
**Education** (Min = 1 / Max = 8)	58.0%	73.5%	+15.5%
**R. Makes Med. Decisions for Children** (No = 0 / Yes = 1)	64.0%	74.0%	+10.0%
**News Consumption** (Min = 0 / Max = 7)	57.6%	70.2%	+12.6%
**Proximity (“strongly distrust” only)** (Min = 52 / Max = 1,057)	63.9%	15.4%	- 48.5%

All other variables held at their observed values. Results based on estimates in Model 2.

While the substantive effects of these control variables are meaningful, they are notably weaker than the influence of our main predictor (proximity to a recent measles outbreak). Focusing only on respondents with low levels of trust in government medical experts, the estimated probability of falling above the midpoint on our dependent variable decreases by more than 48 percent as a subject moves from the minimum proximity value (52 miles) to the maximum (1,057 miles). More specifically, for low-trust individuals who live far away from a recent measles outbreak, the predicted margin of expressing favorable vaccination attitudes is only 15.4 percent. As such, the calculations presented in this section demonstrate that the interaction of trust and proximity creates a substantively meaningful causal effect that should be noted by scholars and public health practitioners alike.

## Discussion and conclusion

The main goal of this paper was to explore vaccination attitudes with regards to measles. In particular, we investigated the effects of (1) spatial proximity to a recent measles outbreak and (2) trust in government medical experts on individual-level attitudes. Building on Construal Level Theory (CLT), we formulated two testable hypotheses. First, we expected that individuals who live closer to a recent measles outbreak should have more favorable attitudes towards vaccinations for this disease than respondents who live farther away. Second, we predicted that this relationship would be moderated by people’s level of trust in government medical experts. Our statistical analysis was based on original survey data, collected in early 2017. Empirically, we found that proximity to a recent outbreak has no independent statistical effect on respondent attitudes. However, as expected, there is a significant interactive relationship between proximity and trust. In particular, low-trust respondents become noticeably less likely to express support for immunizations at increasing distance from a recent outbreak.

Our study has a number of limitations that provide potential avenues for future research. First, our statistical analysis is based entirely on cross-sectional survey data. Thus, we were unable to assess to what extent *recency* of a particular disease outbreak influences vaccination attitudes. However, Construal Level Theory (CLT) explicitly predicts that *both* spatial and temporal proximity affect an individual’s cognitive engagement with a given social phenomenon. As such, more time-series research designs are needed to investigate the temporal dynamics of vaccination attitudes. Second, in this study, we held the type of disease constant by focusing specifically on measles. Moving forward, scholars should explore whether public attitudes vary across diseases with different attributes. For example, it is possible that the severity of a given disease (expressed in terms of morbidity and mortality) moderates the effects of both trust and proximity.

Additionally, disease attributes such as mode of transmission could also explain vaccination attitude. Measles is not only one of the most contagious infectious diseases, but it is transmitted by direct contact *and* via airborne spread. Due to modern social mobility, one’s likelihood of exposure to measles is perhaps much higher than say for a water-borne infectious disease such as typhoid fever, cholera, leptospirosis and hepatitis A resulting from flooding contained to a geographic locale, or a vector-borne diseases such as dengue, Zika or chikungunya prevalent in South American and African countries but not in the U.S. While measles is a disease with a more global reach, other diseases are more geographically contained and therefore there might be a differential impact on vaccination attitudes depending on the particular disease and mode of transmission.

Another consideration is the impact that vaccination laws and requirements have on disease attitudes. Not only do vaccination laws and allowances for exemptions vary across the U.S. states, they also vary from country to country. With differences in both state and national vaccination requirements, we might see a differential impact on vaccination attitudes resulting from an outbreak. That is, individuals in states or countries with strict vaccine laws and little ability for exemptions might respond differently than those who live in states or countries with more lenient vaccine requirements and easily obtainable exemptions. Moreover, effects of laws and exemptions might also interact with size and density of a particular country/state or geographic proximity to a political border between two countries/states, especially one that might have more lenient vaccination and exemption laws. The U.S. is geographically large. However, sampling a more geographically contained population, from a confined region, state, or small country, might show that geographic proximity matters in a way that was not captured with the national sample in the U.S. that was used herein.

In order to test these possibilities, more original survey research is necessary. As well, subsequent studies should investigate potential cross-disease spillover-effects. In this paper, we studied if/how a person’s distance from a recent measles outbreak affects attitudes with regards to that particular disease. However, it is possible that a particular public health crisis raises the salience and perceived danger of various infectious diseases simultaneously, thereby affecting mass attitudes more broadly. Given these considerations, the literature on individual-level vaccination behavior constitutes fertile ground for future research.

There are some practical implications for our study. In particular, distrust of governmental medical experts demonstrates a significant and negative effect on vaccination attitudes. This finding, however, does not necessarily mean that distrust across medical experts is uniform. It is quite possible that other medical experts, such as primary care physicians, school nurses, or midwives instill much greater trust in the minds of the public than do government medical experts and therefore might be reliable resources for encouraging vaccination behavior. For example, two recent studies in Italy have found that vaccine hesitancy is more common among those who do not trust pediatricians [38], among those who receive their information through mass-media [39], and among those who agree with political leaders who oppose vaccination [39]. Furthermore, over half of the parents surveyed expressed a desire for more information about childhood vaccinations [38]. Both studies suggest that these considerations emphasize the need and opportunity for health care workers to improve public trust in scientific information about childhood vaccinations. Although Italy differs from the U.S. in many respects, comparison of Parent Attitudes and Childhood Vaccines scores between the two countries are very similar, despite differences between methods of recruitment and use of measurement [38]. So, while our results regarding proximity are perhaps limited to the uniqueness of U.S. geography, we have reason for believing that our results and practical implications pertaining to trust are generalizable beyond the U.S.

We also see that demographic differences demonstrate significant impact on vaccine likelihood. In particular, individuals with children are more likely to vaccinate than those without children. It could be that parents, with their regularly scheduled doctor’s visits, children’s immunization schedules, and concern about their child’s welfare, are also more engaged in their own immunization health. To even increase the likelihood of vaccination of parents, medical practitioners could, while administering vaccinations to children, also offer vaccinations to the adult caregivers. The parents would then not need an extra trip to the doctor or a separate trip to the pharmacy to receive vaccinations. Also, medical practitioners could perhaps find opportunities to administer vaccinations outside of medical appointments. Workplace vaccination programs might be a good solution for particular sub-populations who are less likely to vaccinate, such as men, older individuals, and those without children. Finally, the news media could take an active role in communicating health information regarding infectious diseases and vaccinations protocols. The news media could be a formidable agent in influencing health and vaccine attitudes, intentions, and behaviors.

## Supporting information

S1 File Replication data. (DTA)

S2 File Replication commands. (TXT)

S1 Table Sample characteristics. (DOCX)

S2 Table Robustness checks (using base indicators). (DOCX)

S1 Text Questionnaire. (DOCX)

## References

[1] Wadman M, You J. The vaccine wars. Science (80-). 2017;356: 364–365. 10.1126/science.356.6336.364 28450592

[2] Olive JK, Hotez PJ, Damania A, Nolan MS. The state of the antivaccine movement in the United States: A focused examination of nonmedical exemptions in states and counties (PLoS medicine (2018) 15 6 (e1002578)). PLoS Med. 2018;15: e1002616 10.1371/journal.pmed.1002616 29894470 PMC5997312

[3] Larson HJ, Jarrett C, Eckersberger E, Smith DMD, Paterson P. Understanding vaccine hesitancy around vaccines and vaccination from a global perspective: A systematic review of published literature, 2007–2012. Vaccine. Elsevier Ltd; 2014;32: 2150–2159. 10.1016/j.vaccine.2014.01.081 24598724

[4] Dales LG, Kizer KW, Rutherford GW, Pertowski CA, Waterman SH, Woodford G. Measles epidemic from failure to immunize. West J Med. 1993;159: 455–464. 8273330 PMC1022280

[5] Wu S, Yang P, Li H, Ma C, Zhang Y, Wang Q. Influenza vaccination coverage rates among adults before and after the 2009 influenza pandemic and the reasons for non-vaccination in Beijing, China: A cross-sectional study. BMC Public Health. 2013;13 10.1186/1471-2458-13-636 23835253 PMC3708734

[6] Poland GA. The 2009–2010 influenza pandemic: Effects on pandemic and seasonal vaccine uptake and lessons learned for seasonal vaccination campaigns. Vaccine. 2010;28: 3–13. 10.1016/j.vaccine.2010.08.024 20713258

[7] Borse RH, Shrestha SS, Fiore AE, Atkins CY, Singleton JA, Furlow C, et al Effects of vaccine program against pandemic influenza A (H1N1) Virus, United States, 2009–2010. Emerg Infect Dis. 2013;19: 439–448. 10.3201/eid1903.120394 23622679 PMC3647645

[8] Bolton P, Holt E, Ross A, Hughart N, Guyer B. Estimating vaccination coverage using parental recall, vaccination cards, and medical records. Public Health Rep. 1998;113: 521–6. Available: http://www.ncbi.nlm.nih.gov/pmc/articles/PMC1308435/%5Cnhttp://www.pubmedcentral.nih.gov/articlerender.fcgi?artid=1308435&tool=pmcentrez&rendertype=abstract 9847923 PMC1308435

[9] Driedger SM, Maier R, Furgal C, Jardine C. Factors influencing H1N1 vaccine behavior among Manitoba Metis in Canada: A qualitative study. BMC Public Health. 2015;15: 1–15. 10.1186/1471-2458-15-1 25884562 PMC4334920

[10] Hulsey E, Bland T. Immune overload: Parental attitudes toward combination and single antigen vaccines. Vaccine. Elsevier Ltd; 2015;33: 2546–2550. 10.1016/j.vaccine.2015.04.020 25891399

[11] Martin LR, Petrie KJ. Understanding the dimensions of anti-vaccination attitudes: The vaccination attitudes examination (VAX) scale. Ann Behav Med. Annals of Behavioral Medicine; 2017;51: 652–660. 10.1007/s12160-017-9888-y 28255934

[12] Wooten KG, Luman LT, Barker LE. Socioeconomic factors and persistent racial disparities in childhood vaccination. Am J Health Behav. 2007;31: 434–445. 10.5555/ajhb.2007.31.4.434 17511578

[13] Shawn D, Gold R. Survey of parents’ attitudes to the recommended Haemophilus influenzae type b vaccine program. CMAJ. 1987;136: 1038–1040. Available: http://www.ncbi.nlm.nih.gov/entrez/query.fcgi?cmd=Retrieve&db=PubMed&dopt=Citation&list_uids=3497703 3494496 PMC1492566

[14] Salmon DA, Moulton LH, Omer SB, DeHart MP, Stokley S, Halsey NA. Factors associated with refusal of childhood vaccines among parents of school-aged children: A case-control study. Arch Pediatr Adolesc Med. 2005;159: 470–476. 10.1001/archpedi.159.5.470 15867122

[15] Smith P, Chu S, Barker L. Children who have received no vaccines: who are they and where do they live? Ped. 2004;114: 187–795. 10.1542/peds.114.1.187 15231927

[16] Vannice KS, Salmon DA, Shui I, Omer SB, Kissner J, Edwards KM, et al Attitudes and beliefs of parents concerned about vaccines: impact of timing of immunization information. Pediatrics. 2011;127: S120–S126. 10.1542/peds.2010-1722R 21502250 PMC4536578

[17] Timmermans DRM, Henneman L, Hirasing RA, van der Wal G. Attitudes and risk perception of parents of different ethnic backgrounds regarding meningococcal C vaccination. Vaccine. 2005;23: 3329–3335. 10.1016/j.vaccine.2005.01.075 15837239

[18] Suryadevara M, Bonville CA, Ferraioli F, Domachowske JB. Community-centered education improves vaccination rates in children from low-income households. Pediatrics. 2013;132: 319–325. 10.1542/peds.2012-3927 23837177

[19] Liberman N, Trope Y. The psychology of transcending the here and now. Science (80-). 2008;322: 1201–1205. 10.1126/science.1161958 19023074 PMC2643344

[20] Spence A, Poortinga W, Pidgeon N. The psychological distance of climate change. Risk Anal. 2012;32: 957–972. 10.1111/j.1539-6924.2011.01695.x 21992607

[21] Langford IH, Georgiou S, Bateman IJ, Day RJ, Turner RK. Public perceptions of health risks from polluted coastal bathing waters: A mixed methodological analysis using cultural theory. Risk Anal. 2000;20: 691–704. 10.1111/0272-4332.205062 11110215

[22] Peters E, Slovic P. The role of affect and worldviews as orienting dispositions in the perception and acceptance of nuclear power. J Appl Soc Psychol. 2006;26: 1427–1453. 10.1111/j.1559-1816.1996.tb00079.x

[23] Williams BL, Brown S, Greenberg M, Kahn MA. Risk perception in context: The Savannah River site stakeholder study. Risk Anal. 1999;19: 1019–1035. 10.1023/A:1007095808381 10765444

[24] Harclerode MA, Lal P, Vedwan N, Wolde B, Miller ME. Evaluation of the role of risk perception in stakeholder engagement to prevent lead exposure in an urban setting. J Environ Manage. Elsevier Ltd; 2016;184: 132–142. 10.1016/j.jenvman.2016.07.045 27477350

[25] Chandran S, Menon G. When a day means more than a year: Effects of temporal framing on judgments of health risk. J Consum Res. 2004;31: 375 Available: http://proquest.umi.com/pqdweb?did=772623601&Fmt=7&clientId=12010&RQT=309&VName=PQD

[26] Rudisill C, Costa-Font J, Mossialos E. Behavioral adjustment to avian flu in Europe during spring 2006: The roles of knowledge and proximity to risk. Soc Sci Med. Elsevier Ltd; 2012;75: 1362–1371. 10.1016/j.socscimed.2012.06.005 22835921

[27] Zielinski-Gutierrez EC, Hayden MH. A Model for defining West Nile virus risk perception based on ecology and proximity. Ecohealth. 2006;3: 28–34.

[28] Jena Anupam B. and Khullar D. To Increase vaccination rates, share information on disease outbreaks. Harv Bus Rev. 2017; 1–5. Available: https://hbr.org/2017/02/to-increase-vaccination-rates-share-information-on-disease-outbreaks

[29] Rosoff H, John RS, Prager F. Flu, risks, and videotape: Escalating fear and avoidance. Risk Anal. 2012;32: 729–743. 10.1111/j.1539-6924.2012.01769.x 22332702

[30] O’Connor RE, Bord RJ, Fisher A. Rating threat mitigators: Faith in experts, governments, and individuals themselves to create a safer world. Risk Anal. 1998;18: 547–556. 10.1111/j.1539-6924.1998.tb00368.x 9853391

[31] Hamilton LC, Hartter J, Saito K. Trust in scientists on climate change and vaccines. SAGE Open. 2015;5 10.1177/2158244015602752

[32] Vaske JJ, Miller CA, Ashbrook AL, Needham MD. Proximity to chronic wasting disease, perceived risk, and social trust in the managing agency. Hum Dimens Wildl. Routledge; 2018;23: 115–128. 10.1080/10871209.2018.1399317

[33] Byington CL. Vaccines: Can transparency increase confidence and reduce hesitancy? Pediatrics. 2014;134: 377–379. 10.1542/peds.2014-1494 25086161 PMC4187240

[34] Baumgaertner B, Carlisle JE, Justwan F. The influence of political ideology and trust on willingness to vaccinate. PLoS One. 2018;13 10.1371/journal.pone.0191728 29370265 PMC5784985

[35] Survey Sampling International. Global Panel Book. Shelton, CT; 2017.

[36] Fill M-M, Morrow H, Sweat D, Haushalter A, Martin JC, Zerwkh T, et al. Measles uutbreak of unknown source—Shelby County, Tennessee, April to May, 2016. Morbidity and Mortality Weekly Report. 2016. 10.15585/mmwr.mm6538a3 27685014

[37] Venkat H, Kassem A, Su C, Hill C, Timme E, Briggs G, et al. Notes from the field: Measles outbreak at a United States Immigration and Customs Enforcement Facility ― Arizona, May–June 2016 [Internet]. Morbidity and Mortality Weekly Report. 2017. 10.15585/mmwr.mm6620a5 PMC5657873 28542125

[38] Napolitano F, A D’Alessandro, IF Agelillo. Investigating Italian parents’ vaccine hesitancy: A cross-sectional survey. Hum Vaccin Immunother. 2018; 1558–1565. 10.1080/21645515.2018.1463943 29641945 PMC6067864

[39] Bianco A, Mascaro V, Zucco R, Pavia M. Parent perspectives on childhood vaccination: How to deal with vaccine hesitancy and refusal? Vaccine. Elsevier Ltd; 2019;37: 984–990. 10.1016/j.vaccine.2018.12.062 30655175

