# Supplementary material for: The effect of trust and proximity on vaccine propensity
Source: PLoS One. 2019 Aug 28;14(8):e0220658. doi: 10.1371/journal.pone.0220658 (PMC6713324; doi:10.1371/journal.pone.0220658)
Supplement: S1 Table — (DOCX) [file pone.0220658.s003.docx]

*S1 Table: Sample Characteristics*

| **Variable** | **Population Data**  **(Census 2010)** | **Sample**  **Statistics** |
| --- | --- | --- |
| *Age Group* |  |  |
| 18-24 | 13.08% | 11.19% |
| 25-34 | 17.51% | 17.48% |
| 35-44 | 17.51% | 17.48% |
| 45-54 | 19.19% | 19.39% |
| 55-64 | 15.55% | 16.18% |
| 65 or older | 17.17% | 18.28% |
|  |  |  |
| *Gender* |  |  |
| Male | 48.53% | 48.25% |
| Female | 51.47% | 51.75% |
|  |  |  |
| *Income* |  |  |
| Less than 30,000 | 29.00% | 30.37% |
| 30,000 – 49,999 | 19.00% | 19.28% |
| 50,000 – 74,999 | 18.00% | 17.98% |
| 75,000 – 99,999 | 12.00% | 12.09% |
| More than 100,000 | 22.00% | 20.28% |
|  |  |  |
| *Self-reported Ethnicity* |  |  |
| Hispanic or Latino | 16.30% | 16.38% |
| White Alone | 63.70% | 63.54% |
| African-American | 12.20% | 12.29% |
| Asian | 4.70% | 4.70% |
|  |  |  |
| *Region* |  |  |
| Northeast | 18.00% | 17.98% |
| Midwest | 22.00% | 22.28% |
| South | 37.00% | 36.57% |
| West | 23.00% | 23.18% |
