# Supplementary material for: The effect of trust and proximity on vaccine propensity
Source: PLoS One. 2019 Aug 28;14(8):e0220658. doi: 10.1371/journal.pone.0220658 (PMC6713324; doi:10.1371/journal.pone.0220658)
Supplement: S2 Table — (DOCX) [file pone.0220658.s004.docx]

*S2 Table: Robustness Checks (using base indicators)*

| ***Ordered Logistic Regression*** | **Model 1**  **(Low Risk Scenario)** | **Model 2**  **(High Risk Scenario)** |
| --- | --- | --- |
| **Age of Respondent (in years)**  (Min = 18 / Max = 97) | -0.017**  (0.004) | -0.018**  (0.005) |
| **Gender of Respondent**  (Female = 0 / Male = 1) | -0.199*  (0.121) | -0.145  (0.129) |
| **Education Level of Respondent**  (Min = 1 / Max = 8) | 0.089**  (0.040) | 0.143**  (0.043) |
| **Income Level of Respondent**  (Min = 1 / Max = 12) | 0.022  (0.020) | 0.030  (0.021) |
| **Race of Respondent**  (Non-White = 0 / White = 1) | 0.098  (0.149) | 0.249  (0.157) |
| **R. Makes Medical Decisions for at Least one Child**  (No = 0 / Yes = 1) | 0.523**  (0.135) | 0.428**  (0.146) |
| **News Consumption of Respondent (days per week)**  (Min = 0 / Max = 7) | 0.081**  (0.032) | 0.085**  (0.033) |
| **Respondent Proximity to Recent Measles Outbreak**  (Min = 0 / Max = 2,946) | 0.001  (0.001) | 0.001  (0.001) |
| **Trust in Gov. Medical Experts (“Strongly distrust”)**  (Strongly Distrust = 1 / Other = 0) | 0.612  (0.651) | 0.833  (0.673) |
| **Trust in Gov. Medical Experts (“Somewhat distrust”)**  (Somewhat Distrust = 1 / Other = 0) | 0.504  (0.489) | 0.654  (0.513) |
| **Trust in Gov. Medical Experts (“Somewhat trust”)**  (Somewhat Trust = 1 / Other = 0) | 0.575**  (0.276) | 0.757**  (0.300) |
| **Trust in Gov. Medical Experts (“Strongly trust”)**  (Strongly Trust = 1 / Other = 0) | 1.228**  (0.386) | 1.516**  (0.424) |
| **Proximity X Strongly distrust** | -0.003**  (0.001) | -0.003**  (0.001) |
| **Proximity X Somewhat distrust** | -0.001  (0.001) | -0.001*  (0.001) |
| **Proximity X Somewhat trust** | -0.001  (0.001) | -0.001  (0.001) |
| **Proximity X Strongly trust** | 0.001  (0.001) | -0.001  (0.001) |
| **Cut Point 1** | -1.021 (0.342) | -1.022 (0.367) |
| **Cut Point 2** | -0.103 (0.337) | -0.333 (0.360) |
| **Cut Point 3** | 0.763 (0.338) | 0.489 (0.358) |
| **Cut Point 4** | 1.810 (0.343) | 1.480 (0.362) |
| **Number of Observations** | 931 | 933 |
| **Log-Likelihood** | -1368.60 | -1152.72 |
| * = p ≤ 0.10, ** = p ≤ 0.05 |  |  |
