# Supplementary material for: The effect of trust and proximity on vaccine propensity
Source: PLoS One. 2019 Aug 28;14(8):e0220658. doi: 10.1371/journal.pone.0220658 (PMC6713324; doi:10.1371/journal.pone.0220658)
Supplement: S1 Text — (DOCX) [file pone.0220658.s005.docx]

*S1 Text. Survey Questions For Variables Used In Statistical Analysis*

Attitude About Vaccination For Measles (low risk scenario): Suppose you are missing the vaccine for the following diseases but there is no immediate risk of getting infected - how likely or unlikely are you to get vaccinated?

|  | **Very Unlikely** | **Unlikely** | **Neither Likely nor Unlikely** | **Likely** | **Very Likely** | **I Don’t Know** |
| --- | --- | --- | --- | --- | --- | --- |
| Measles | 1 | 2 | 3 | 4 | 5 | 99 |
| …other diseases omitted (not relevant for this project)… | | | | | | |

Attitude About Vaccination For Measles (high risk scenario): Suppose again you are missing the vaccine for the following diseases but now there is an outbreak of that disease in your community - how likely or unlikely are you to get vaccinated?

|  | **Very Unlikely** | **Unlikely** | **Neither Likely nor Unlikely** | **Likely** | **Very Likely** | **I Don’t Know** |
| --- | --- | --- | --- | --- | --- | --- |
| Measles | 1 | 2 | 3 | 4 | 5 | 99 |
| …other diseases omitted (not relevant for this project)… | | | | | | |

Trust in Government Medical Experts: How much do you trust the following sources regarding questions about health?

|  | **Strongly Distrust** | **Somewhat Distrust** | **Neither Trust Nor Distrust** | **Somewhat Trust** | **Strongly Trust** | **I Don’t Know** |
| --- | --- | --- | --- | --- | --- | --- |
| Government medical experts such as Centers for Disease Control and Prevention | 1 | 2 | 3 | 4 | 5 | 99 |
| …other enttites omitted (not relevant for this project)… | | | | | | |

Age: What is your age?

Gender: Are you male or female?

- Male
- Female

News Consumption: During a typical week, how many days do you watch, read, or listen to the news, NOT including sports?

- 0
- 1
- 2
- 3
- 4
- 5
- 6
- 7
- I don’t know

Education: What is the highest level of school you have completed or the highest degree you have received?

- Less than high school (Grades 1-8 or no formal education)
- High school incomplete (Grades 9-11 or Grade 12 with NO diploma)
- High school graduate (Grade 12 with diploma or GED certificate)
- Some college, no degree (includes some community college)
- Two year associate degree from a college or university
- Four year college or university degree/Bachelor’s degree (e.g., BS, BA, AB)
- Some postgraduate or professional schooling, no postgraduate degree (e.g. some graduate school)
- Postgraduate or professional degree, including master’s, doctorate, medical or law degree (e.g., MA, MS, PhD, MD, JD, graduate school)

Income: Last year, that is in 2016, what was your total family income from all sources, before taxes?

- Less than $10,000
- Between $10,000 and $19,999
- Between $20,000 and $29,999
- Between $30,000 and $39,999
- Between $40,000 and $49,999
- Between $50,000 and $59,999
- Between $60,000 and $69,999
- Between $70,000 and $79,999
- Between $80,000 and $89,999
- Between $90,000 and $99,999
- Between $100,000 and $149,999
- More than $150,000

Race: Which of the following describes your race? You can select as many as apply.

- White
- Black or African-American
- Asian or Asian-American
- Some other race

R. Makes Med. Decisions for Children: Do you have children or dependents for whom you make medical decisions?

- Yes
- No

Zip Code (used to calculate Proximity to Recent Measles Outbreak): What is your 5 digit zip code?

_ _ _ _ _
